# Semiochemical baited traps of lepidopteran pests of economic importance can deliver reliable data also on wide range of non-target species: case study in the Hajdúság Region of East Pannonian Lowland (East Hungary)

**DOI:** 10.3897/BDJ.9.e72305

**Published:** 2021-10-29

**Authors:** Tímea Szalárdi, Szabolcs Szanyi, István Szarukán, Miklós Tóth, Antal Nagy

**Affiliations:** 1 University of Debrecen Faculty of Agricultural and Food Sciences and Environmental Management, Institute of Plant Protection, Debrecen, Hungary University of Debrecen Faculty of Agricultural and Food Sciences and Environmental Management, Institute of Plant Protection Debrecen Hungary; 2 Plant Protection Institute, Centre for Agricultural Research, ELKH, Budapest, Hungary Plant Protection Institute, Centre for Agricultural Research, ELKH Budapest Hungary

**Keywords:** fauna, distribution data, Lepidoptera, Carpathian Basin, non-target catches, phenylacetaldehyde, isoamyl alcohol

## Abstract

Lepidopteran assemblages were studied at 16 sampling sites in the Hajdúság Region between 2013 and 2020. Although studies targeted development of synthetic phenylacetaldehyde-based and semi-synthetic isoamyl alcohol-based baits for pest monitoring, traps caught 179 species belonging to the Sphingidae, Thyatiridae, Geometridae, Erebidae and Noctuidae**families. Most species were pests or widely distributed generalists, but there were also many rare habitat specialists, for example, silvicol species, whose appearance was unexpected in the recently less forested region. The specificity of the two bait types tested differed notably both on family and subfamily levels. Semi-synthetic baits performed better and attracted a wide range of noctuids belonging mainly to the Xyleninae and Noctuinae subfamilies, while synthetic phenylacetaldehyde-based lures showed specificity to Plusiinae subfamilies with lower number of sampled species. Our data fill a gap of knowledge since the fauna studied formerly was nearly unknown and brings attention to the alternative use of volatile traps of agricultural pests in faunistical studies.

## Introduction

The Hajdúság is a 60 km long and 20 km wide north-south-orientated region in the eastern part of the Carpathian Lowland (East Hungary), where the varied edaphic and soil conditions and the diverse relief formed a unique and very diverse landscape. Climate of the region is temperately warm and xeric, the yearly mean temperature is about 10-11°C and the mean temperature of the warmest month (July) is between 21.0-21.5°C. The yearly amount of precipitation generally varies between 550 and 600 mm.

Originally, a series of different habitats from xeric sandy grasslands to the wet meadows could be found here and, formerly, this region was one of the most densely forested areas of the Pannonian Lowland (Molnár 2021). During the last century, the intensification of the agriculture and urbanisation with a parallel decrease and fragmentation of natural habitats have dramatically changed the vegetation and habitat structure, soil characteristics and meso-climate of the region (Bíró 2011, Centeri et al. 2012, Szilassi 2017). Due to deforestation and change of species composition of the remaining forests (from oak to acacia, poplar and fir) and expansive cultivation of former meadows and wetlands, nowadays only fragmented and isolated patches of natural-like habitats can be found (Anonymous 1999).

The most characteristic Pannonian saline grasslands are mainly mowed and grazed by cattle or sheep. The northern part is mostly cultivated, since this is one of the most productive agricultural lands of Hungary, where only small, fragmented remains of natural-like habitats can be found mostly on hedges, roadsides and kurgans. The inner part of the south Hajdúság is also mainly cultivated, but it preserves larger saline habitats in the northern and southern margins.

The Lepidoptera fauna of the region is largely unknown. There are only scattered data based on occasional samplings (Baranyi et al. 2006). The neighbouring Hortobágy (Vojnits and Ronkay 1981, Ronkay et al. 1983) and Nyírség (Szanyi et al. 2019) Regions are generally more studied considering other invertebrate taxa (e.g. Nagy and Rácz 2007b, Nagy and Rácz 2007a, Sólymos and Fehér 2005), but their Lepidoptera fauna are less known. In case of Hortobágy, mainly scattered data on some protected species are available despite of the protected status of the area (Baranyi et al. 2006).

Although the general method used in studies on Macroheterocera fauna and assemblages is light-trapping, there are alternative sampling methods, such as use of different baits and direct search of adults. North American, European and even many Hungarian studies have proved that baits containing isoamyl alcohol or phenylacetaldehyde attract not only pest Lepidoptera, but also a large number of other non-target species belonging to the Sphingidae*, *Thyatiridae*,* Geometridae*, *Erebidae*,* Noctuidae and many other moth families (Landolt 2000, Landolt and Alfaro 2001, Landolt and Highbee 2002, Szanyi et al. 2017, Szanyi et al. 2019, Nagy et al. 2014, Tóth et al. 2010, Tóth et al. 2015).

Since 2013 in the Hajdúság, 16 studies have been carried out during development of bisexual lure, targeting mainly the European corn borer (*Ostrinia* *nubilalis*) (Tóth et al. 2016, Tóth et al. 2017), the silver Y moth (*Autographa* *gamma*) (Tóth et al. 2019) and the cotton bollworm (*Helicoverpa* *armigera*) (Tóth et al. 2020). Here, we discuss the non-target Lepidoptera catches of these field studies to provide a checklist of the Macroheterocera fauna and to draw attention to the need for further intensive investigations of the region. Beyond that, the range of attraction and selectivity of different lures was also characterised both at the species and family levels.

## Material and methods

### Sampling

**Figure 1.**
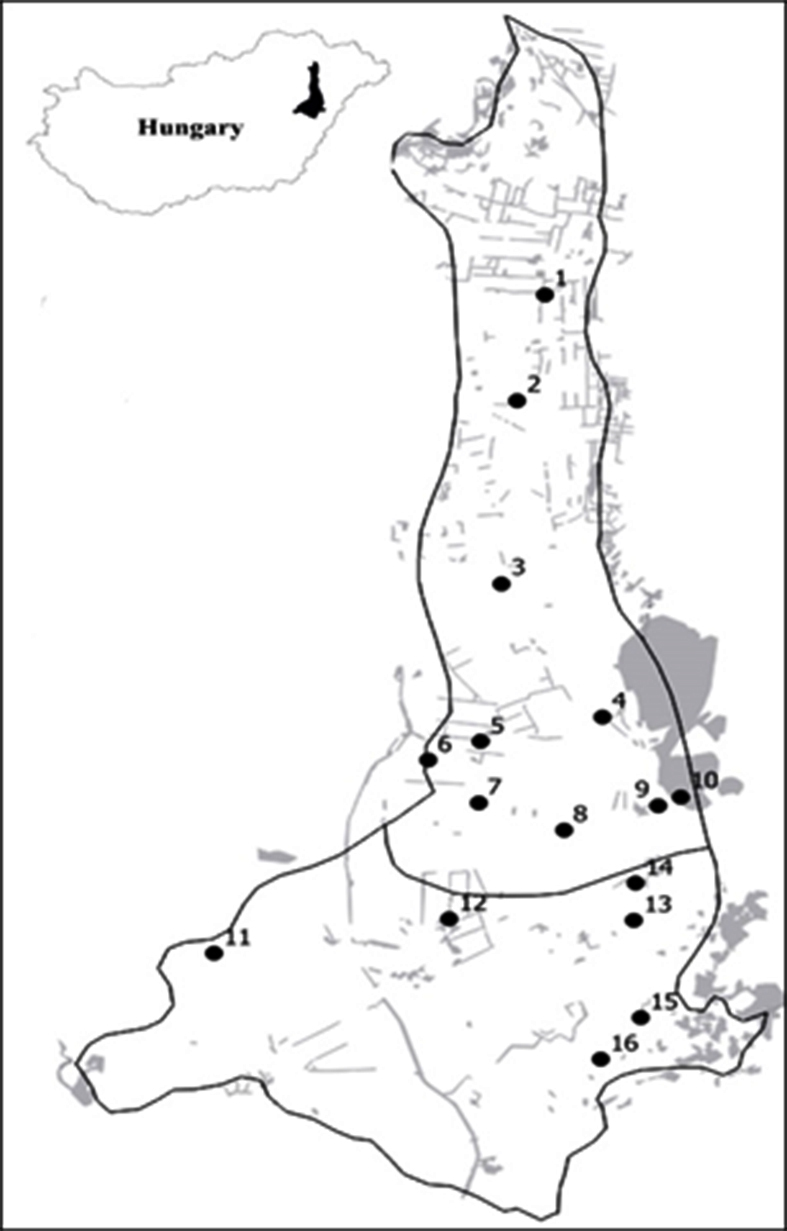
Location of the semiochemical-baited trap sampling sites (black dots) of the Lepidoptera fauna in the Hajdúság in East Hungary. Black line: border of region and subregions, grey polygons: forested areas, forest patches and larger forest belts. Site numbers refer to the numbers used in Table 1.

Samplings were carried out at 16 sites of the Hajdúság Region in the surroundings of Balmazújváros, Debrecen, Derecske, Hajdúböszörmény, Hajdúdorog, Hajdúnánás, Hajdúszuboszló, Nádudvar Cities and Sáránd Village between 2013 and 2020 (Fig. 1, Table 1). Traps were operated during the vegetation period generally from May and June to September and October or sometimes to November (Table 1).

Samplings were made using CSALOMON VARL+ funnel traps (Plant Protection Institute, CAR, ELKH, Budapest, Hungary). During the studies, phenylacetaldehyde-based synthetic (PHEN) and isoamyl alcohol-based semi-synthetic (IamOH) lures were used (Table 2) (Nagy et al. 2014, Szanyi et al. 2017, Szanyi et al. 2019, Tóth et al. 2010, Tóth et al. 2015).

In the case of iso-amyl alcohol-based lures, polypropylene tubes with 4 ml capacity were used as dispensers (Tóth et al. 2015). Mixtures were administered on dental rolls inside tubes with a small (4 mm diam.) hole at the bottom of the tube, opening at trap deployment. The upper, larger opening of the tube was closed.

In the case of phenylacetaldehyde-based lures, polyethylene bag dispensers (Tóth et al. 2002) were used. These lures all contained phenylacetaldehyde as base component and one or more other potentially synergistic compounds (refer to Table 2), depending on the target lepidopteran pest (e.g. *Ostrinia* *nubilalis*, *Autographa* *gamma*, *Helicoverpa* *armigera* etc.) (Tóth et al. 2019, Tóth et al. 2016, Tóth et al. 2020, Tóth et al. 2017).

The moths caught were killed by Vaportape II insecticide strip developed especially for use in insect traps (10% 2,2 dichlorovinyl dimethyl phosphate). This insecticide does not affect the attractivity of the bait and quickly kills insects fallen into the trap. All bait types tested were exposed in five replicates at each site, 15-20 m distant from each other, at 1.8-2 m elevations. Traps were fixed on branches of trees or on stakes. The baits were changed every four weeks, while the traps were checked and the caught insects were removed twice a week.

### Data analysis

The collected material was deep-frozen and stored until identification. The sampled Noctuidae taxa were identified to species level if possible, according to Varga 2012). The taxonomic list follows the system by Lafontaine and Schmidt (Lafontaine and Schmidt 2010) with modifications of Zahiri et al*.* (Zahiri et al. 2012). In the case of subdivision of faunal elements and ecological types (so-called faunal components), the methodology of Varga et al. (Varga et al. 2004) was followed. The data on hosts (herbaceous vs. woody food plants) were obtained mostly from standard works on European moths (e.g. Fibiger et al. 2009, Fibiger 2010, Hacker et al. 2002, László et al. 2001). In the case of the protection status, the country-wide list of protected species was followed (termeszetvedelem.hu 2020). Species that were mentioned as agricultural or forestry pests in literature were listed separately.

The fauna studied was characterised by total and mean local species richness and percentage (%) of taxa caught (families and subfamilies), pests, species with different level of conservation value, feeding- and faunal types and faunal compositions. The local rarity of the species was established as a spatial constancy. Since the lures used showed different range and selectivity, the values were calculated for the two lure types separately and were finally established as the mean of these two values.

The range of attraction and selectivity of different lures was compared, based on total and mean number of species caught, ratios of the above-mentioned variables and number of differential species attracted with only synthetic or semi-synthetic lures. Means of these variables were compared with the Mann-Whitney U test since the assumptions of the parametric test were not fulfilled. Tests were carried out with Statistica 7 (StatSoft Inc. 2004).

## Results

In the 18 experiments carried out at 16 sites, traps caught a little more than 36,000 Macroheterocera specimens belonging to 179 species of five families (Sphingidae*, *Thyatiridae*,* Geometridae*, *Erebidae**and Noctuidae). The largest part (139 species, 77.7%) belonged to the Noctuidae family and the Erebidae was the second most species-rich taxa with 22 species. Amongst noctuids, Xyleninae, Hadeninae and Noctuinae were the most species-rich subfamilies (Table 3).

The total number of species attracted by phenylacetaldehyde-based (PHEN) and isoamyl alcohol-based (IamOH) lures were similar, but the mean number of species sampled was significantly higher in experiments with isoamyl alcohol-based lures. These traps generally caught more species of Thyatiridae, Erebidae and Noctuidae families and Xyleninae, Hadeninae, Noctuinae and Acronictinae subfamilies (Table 3). Contrarily, the mean number of Geometridae and Plusiinae (Noctuidae) species was higher in experiments with phenylacetaldehyde-based lures, but the difference was significant only in case of Plusiinae.

Considering the ratio of species belonging different families and Noctuidae subfamilies the mean ratio of Erebidae and Thyatiridae family and Xyleninae subfamily were significantly higher in experiments with isoamyl alcohol-based lures, while the ratio of Geometridae and Plusiinae (Noctuidae) species was significantly higher where phenylacetaldehyde-based baits were used (Table 3). In the case of other taxa, the differences were not significant. Considering subfamilies with less than 5 caught species, Cuculiinae (4:1) and Bryophilinae (2:1) were mainly attracted by phenylacetaldehyde-based lures and two Oncocnemidinae species were attracted by only this bait type. In contrast, the only Psaphidinae species caught, *Allophyes* *oxyacanthae*, was found only in isoamyl alcohol-based traps (see Suppl. material 1).

There were 37 different species that were caught with only semi-synthetic and 49 with only synthetic lures. The other 93 species were attracted with both types of bait.

Traps caught two protected species and no strictly protected species were sampled. The number of the faunistically important and/or geographically interesting vulnerable species was relatively low (16 species in total) and their ratio was under 10%. Isoamyl alcohol-based lures attracted much more vulnerable species considering both number and ratio of these species (Table 4).

Amongst the species caught, 31 (17.3%) could be regarded as agricultural pests. The mean number of pest species was significantly higher in the case of isoamyl alcohol-based lures, but their percentage was higher in the case of experiments with phenylacetaldehyde-based lures, although this difference was not significant (Table 4).

More of the species caught were herbaceous rather than woody plant feeders. The mean number of species feeding on arboreal plants was much higher in experiments with isoamyl alcohol-based lures. The mean number of species feeding on herbaceous plant and other alternative food sources (e.g. litter, lichen etc.) did not differ significantly and was also higher when isoamyl alcohol-based lures were used.

In contrast, the percentage of herbaceous feeder species was higher in experiments with phenylacetaldehyde-based traps, while arboreal feeders were more common in isoamyl alcohol-based traps and both differences were significant (Table 4).

In the sampled fauna, Euro-Siberian species showed the highest ratio (53.6%). The other two largest groups were formed by Holo-Mediterranean (24.6%) and Boreo-Continental (10.1%) species. Considering the ratio of species belonging to different faunal types, isoamyl alcohol-based lures showed significant specificity to Holo- and Ponto-Mediterranean species groups, while phenylacetaldehyde-based baits were specific to Holarctic and Euro-Siberian faunal types. Beyond that, the mean ratio of the remaining four faunal types was higher in experiments with isoamyl alcohol-based traps, but these differences were not significant (Table 5).

Most of the caught species were eurytopic. The ratio of different woodland species (Quercetal, Silvicol and Populo-Salicetal) and the relative share of steppic elements were also high. Phenylacetaldehyde-based lures generally attracted significantly higher ratios of species belonging to Altoherbosa and Eurytopic ecological types and isoamyl alcohol-based lures showed significant specificity to the arboreal, such as Populo-Salicetal, Silvicol and Quercetal ecological types. The mean ratios of Arundiphilous, Betulo-Alnetal, Psammophilous and Lichenophagous species were higher, while ratios of Migratory, Steppic, Mesophilous and Marshy ecological types were lower in the course of using isoamyl alcohol-based lures, but these differences were not significant (Table 6).

Based on the spatial constancy, the five most common species of the region were *Hadula* *trifolii*, *Agrotis* *segetum*, *Noctua* *pronuba*, *Macdunnoughia* *confusa* and *Lacanobia* *oleracea* which are all widely distributed eurytopic pest species. These species are generally distributed in the northern temperate zone and feed mainly on herbaceous plants. Amongst arboreal species, only *Catocala* *nupta* and *Agrochola* *circellaris* and the silvicol *Mesapamea* *secalis* and *Thalpophila* *matura* showed large spatial constancy (Table 7).

**Figure 2.**
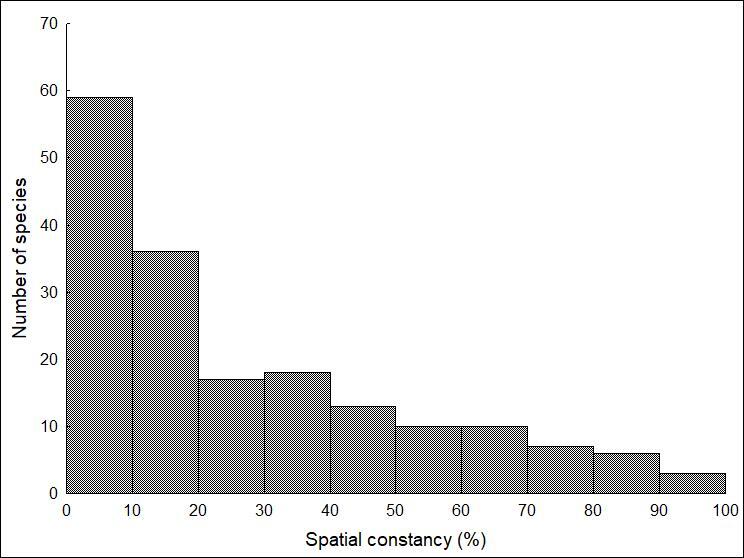
Histogram of mean spatial constancy of Macroheterocera species sampled at 16 sites in the Hajdúság between 2013 and 2020 with different types of semiochemical-baited traps developed against pest species of Lepidoptera.

A considerable part of the species was especially rare in the studied area. Nearly 60 species showed ≤ 10% mean spatial constancy in the studied period. Amongst them were some rare and faunistically vulnerable, mainly arboreal species, such as *Rhizedra* *lutosa*, *Blepharita* *satura*, *Agrochola* *lota*, *Eucarta* *amethystine* and the protected *Lithophane* *semibrunnea* and, additionally, there was another 36 species with a value of spatial constancy between 10 and 20 (Fig. 2).

## Discussion

During development of phenylacetaldehyde-based synthetic and isoamyl alcohol-based semi-synthetic baits and traps, a large part of the formerly unknown Macroheterocera fauna of the Hajdúság Region was revealed. In this relatively small region, occurrence and distribution of 179 species of Macroheterocera were characterised based on > 36,000 specimens. From the neighbouring South Nyírség Region, which is a more forested and less intensively cultivated region with higher habitat diversity, 226 species were reported with use of the same volatiles, but lower sampling intensity (with four experiments) (Szanyi et al. 2019). The high number of species caught reflected the wide attractivity of the tested volatiles (Nagy et al. 2014, Nagy et al. 2015, Szanyi et al. 2017, Szanyi et al. 2019, Szanyi et al. 2020, Tóth et al. 2010).

The largest part of the sampled species belonged to the Noctuidae**family and about 26.0% of the Hungarian fauna were detected in the studied region (Kádár et al. 2010). In the case of Thyatiridae**and Erebidae, this ratio was 30.0% and 16.2%, respectively, thus the used semiochemicals can be used in studies targeting mainly these families. In the case of Sphingidae and Geometridae, only 5.0% and 3.5% of the Hungarian fauna were caught (Varga 2012).

Most of the species feed on herbaceous plants, but traps caught many arboreal feeders. The majority of the fauna consisted of widely distributed Euro-Siberian species, but the ratio of the southern elements (e.g. Holo-Mediterranean species) was also notable. In the fauna studied, the eurytopic species were dominant. The relatively high mean ratio of the arboreal types, such as Silvicol, Quercetal and Populo-Salicetal species (in total, 37.4%), was unexpected considering the recent habitat structure of the region. This high ratio shows that the remaining patches of forest and planted forest belts and alleys could maintain relatively diverse Macroheterocera assemblages. This species-rich arboreal fauna can serve as a basis for further habitat and landscape reconstruction of forested areas.

Although the lures tested were dedicated to attracting mainly lepidopteran pests, they attracted a wide range of species. Generally, about one third (32.6 ± 5.9%) of the sampled species were pests. Nagy et al. (Nagy et al. 2015) found that the environmental risk caused by non-target catches of protected species depended on the habitat structure of the sampling sites. In our case, traps working in mainly agricultural land had no real environmental risk since they caught two protected species with less than five specimens. The use of these attractants and traps in habitats with high conservation value needs further investigations.

The tested phenylacetaldehyde-based and isoamyl alcohol-based lures showed different effect range and selectivity at the level of families and subfamilies and also considering feeding type, faunal element and ecological types of species. Former studies showed that isoamyl alcohol-based semi-synthetic lures can attract a wider range of Macroheterocera species and they are selective to Noctuinae and Xyleninae subfamilies. Contrary to this, phenylacetaldehyde-based lures are selective for Plusiinae and Heliothinae subfamilies (Nagy et al. 2014, Nagy et al. 2015, Szanyi et al. 2017, Szanyi et al. 2019, Szanyi et al. 2020). In this study, the selectivity of isoamyl alcohol-based lures and the selectivity of phenylacetaldehyde-based lures to the Plusiinae species could be proven. Phenylacetaldehyde-based lures were specific to herbaceous feeders, while isoamyl alcohol-based lures were specific to species that feed on arboreal plants. Although the eurytopic species can be effectively sampled with both types of lures, in forestry, the use of isoamyl alcohol-based lures can be recommended due to its specificity to arboreal species. In forestry, such traps can complement and, in some cases (e.g. *Conistra* sp. and *Orthosia* sp., unpublished data of the authors), substitute for the use of light-traps.

Based on our recent and earlier experiments using semiochemical-baited traps, especially in case of simultaneous use of synthetic phenylacetaldehyde-based and semi-synthetic isoamyl alcohol-based baits, the use of these baits can serve as a cheap and very effective method for collecting dsitribution data of many Thyatiridae, Erebidae and Noctuidae species in plant protection and forestry monitoring. Although it may have some environmental risk because of non-target catches of valuable and even protected species, with due care, it can be used also in faunistical studies.

## Supplementary Material

BC8096C9-5F28-5B0D-8B24-6778AE2F188B 10.3897/BDJ.9.e72305.suppl1 Supplementary material 1 Check list of species of Macroheterocera sampled in the Hajdúság Region (East Hungary) with synthetic and semi-synthetic volatile traps between 2013 and 2019 by families, with their food preference, state, faunal and ecological types and distribution in the 16 studied sites. Data type Species listSpecies list Brief description Check list of species of Macroheterocera sampled in the Hajdúság Region (East Hungary) with synthetic and semi-synthetic volatile traps between 2013 and 2019 by families, with their food preference, state, faunal and ecological types and distribution in the 16 studied sites.
A = Arboreal, H = Herbaceous, O = other; Pe = pest, Pr = protected, V = vulnerable; As = Asian, Bor = Boreo, Cas = Caspian, Cont = Continental, Eu = euro, Ex = Extra, Hol = Holarctic, Holo = Holo, M = Manchurian, Med = Mediterranean, Pal = Palearctic, Sib = Siberian, W = West; Altoh = Altoherbosa, Around = Arindinaceous, Bet-Aln = Betulo-Alnetal, Eury = Eurytopic, Lich = Lichenophagous, Marshy = Marshy and marshy woods, Mesop = Mesophilous, Querc = Quercetal, Pop-Sal = Populo-Salicetal, Psamm = Psammophilous, Silv = Silvatic. File: oo_571646.xlsx https://binary.pensoft.net/file/571646 T. Szalárdi – Sz. Szanyi – I. Szarukán. – M. Tóth – A. Nagy.

## Figures and Tables

**Table 1.** Data of the experiments (code of site, location, date, habitat type and baits tested) carried out with semiochemical-baited traps of lepidopteran pests in the Hajdúság between 2013 and 2020. IamOH: isoamyl alcohol-based, PHEN: phenylacetaldehyde-based.

**Site**		**GPS: N**	**GPS: E**	**Habitat**	**Start**	**End**	**Volatile**
1	Hajdúdorog	47°55.4'	21°29.9'	mixed agric.	03.06.2018	31.10.2018	PHEN
2	Hajdúdorog	47°50.8'	21°28.5'	arable land	28.05.2018	29.08.2018	PHEN
3	Hajdúböszörmény	47°42.8'	21°27.7'	arable land	02.07.2016	30.10.2016	PHEN
4	Hajdúböszörmény	47°37.0'	21°33.1'	mixed agric.	10.06.2016	06.09.2016	PHEN
5	Balmazújváros	47°36.2'	21°26.4'	mixed agric.	08.07.2014	22.11.2014.	IamOH
6	Balmazújváros	47°35.0'	21°23.7'	mixed agric.	08.06.2013	06.10.2013.	PHEN
7	Debrecen-Látókép	47°33.2'	21°26.4'	arable land	01.06.2015	03.09.2015	PHEN
8	Debrecen-Ondód	47°32.0'	21°31.1'	arable land	02.07.2013	02.11.2013	IamOH
9	Debrecen	47°33.1'	21°36.1'	suburban	20.05.2016	16.09.2016	both
10	Debrecen	47°33.5'	21°37.3'	suburban	06.06.2017	01.11.2017	PHEN
11	Nádudvar	47°26.6'	21°12.3'	mixed agric.	27.05.2018	09.09.2018	PHEN
12	Hajdúszoboszló	47°28.1'	21°24.9'	mixed agric.	28.05.2016	09.09.2016	PHEN
13	Debrecen-Szepes	47°28.0'	21°34.8'	arable land	24.05. 2018	23.09.2018	both
14	Debrecen-Szepes	47°29.6'	21°34.9'	arable land	17.07.2013	22.10.2013	PHEN
15	Sáránd	47°23.7'	21°35.1'	mixed agric.	09.06.2020	24.09.2020	PHEN
16	Derecske	47°21.9'	21°33.0'	mixed agric.	01.06.2016	05.10.2016	IamOH

**Table 2.** Semiochemical lures tested in field experiments in the Hajdúság between 2013 and 2020 with the number of treatments (Tre) and traps (Tra). PHE = phenylacetaldehyde, 4MOH = 4-methoxyphenethyl alcohol, AN = anethol, EU = eugenol, BA = benzyl acetate, MeSa = methyl salicylate, NH4A = ammonium acetate, BAL = benzaldehyde, IaOH = isoamyl alcohol, AcA = acetic acid, RV = red vine, RVE = red vine extract, Be = Beer (lager), BeE = beer (lager) extract.

				**Synthetic compounds**									**Organic comp.**			
**Site**	**Tra**	**Tre**	PHE	4MOH	AN	EU	BA	MeSa	NH4A	BAL	IaOH	AcA	RV	RVE	Be	BeE
1	20	4	+			+										
2	25	5	+	+					+	+						
3	10	2	+		+	+	+									
4	25	5	+	+												
5	15	3									+	+	+			
6	10	2	+													
7	35	7	+	+												
8	25	5									+	+	+	+	+	+
9	20	4	+	+	+	+	+				+	+	+			
10	10	2	+			+		+								
11	25	5	+	+					+	+						
12	25	5	+	+												
13	10	2	+	+							+	+	+			
14	15	3	+		+											
15	15	3	+	+												
16	5	1									+	+	+			

**Table 3.** Total and mean number of species (species/experiment) caught by different types of semiochemical baited traps in the Hajdúság and percentage (%) of species of families of Macroheterocera (bold letters) and of subfamilies of Noctuidae. Category of "other Noct." (other subfamilies of Noctuidae) shows summarised data of Noctuidae subfamilies with < 5 species in the whole sample: Acontiinae, Cuculliinae, Amphipyrinae, Bryophilinae, Condicinae, Oncocnemidinae, Metoponiinae and Psaphidinae. Small letters show significant differences between bait types, based on the Mann-Whitney U test (p < 0.05). IamOH: isoamyl alcohol-based, PHEN: phenylacetaldehyde-based.

	**Species**	**Species/exp.**	**Species**		**Species/exp.(± SD)**	
**Volatile**		**(± SD)**	**IamOH**	**PHEN**	**IamOH**	**PHEN**
	**Number of species**					
Total	179	45.7 (18.7)	130	142	66.0 (11.4) a	37.9 (14.7) b
**Erebidae**	22	4.1 (3.4)	15	17	7.6 (2.6) a	2.7 (2.6) b
**Geometridae**	14	1.7 (1.6)	3	14	0.6 (0.9)	2.1 (1.7)
**Sphingidae **	1	0.1 (0.2)	1	0	0.2 (0.4)	0.0 (0.0)
**Thyatiridae **	3	0.5 (0.9)	3	1	1.6 (0.9) a	0.1 (0.1) b
**Noctuidae**	139	39.4 (15.4)	108	110	56 (10.1) a	33.1 (12.0) b
Xyleninae	56	12.4 (9.4)	49	38	24.2 (8.1) a	7.8 (4.7) b
Hadeninae	25	8.2 (3.0)	17	23	10.4 (1.1) a	7.4 (3.0) b
Noctuinae	20	7.0 (3.8)	18	15	10.8 (3.6) a	5.5 (2.6) b
Plusiinae	8	4.3 (2.2)	5	7	2.0 (1.6) a	5.2 (1.7) b
Heliothinae	6	1.5 (1.0)	4	6	1.8 (0.8)	1.4 (1.1)
Acronictinae	5	0.9 (1.0)	4	4	2.0 (1.0) a	0.5 (0.7) b
other Noct.	19	4.6 (2.3)	12	17	5.2 (1.3)	4.3 (2.6)
	**Ratio %**					
**Erebidae**	12.3	7.7 (5.5)	11.5	12	11.7 (4.0) a	6.2 (5.3) b
**Geometridae**	7.8	4.1 (3.8)	2.3	9.9	0.8 (1.2) a	5.3 (3.7) b
**Sphingidae **	0.6	0.1 (0.3)	0.8	0	0.3 (0.6)	0.0 (0.0)
**Thyatiridae **	1.7	0.8 (1.3)	2.3	0.7	2.4 (1.2) a	0.1 (0.4) b
**Noctuidae**	77.7	87.4 (6.5)	83.1	77.5	84.8 (3.5)	88.4 (7.2)
Xyleninae	31.3	24.6 (11.0)	37.7	26.8	36.3 (8.8) a	20.1 (8.1) b
Hadeninae	14	18.8 (5.4)	13.1	16.2	16 (1.9)	19.9 (6.0)
Noctuinae	11.2	15.1 (5.6)	13.8	10.6	16.1 (3.4)	14.7 (6.3)
Plusiinae	4.5	11.9 (8.8)	3.8	4.9	3.4 (3.3) a	15.2 (8.0) b
Heliothinae	3.4	3.4 (2.6)	3.1	4.2	3.0 (2.0)	3.6 (2.8)
Acronictinae	2.8	1.8 (1.7)	3.1	2.8	2.9 (1.1)	1.4 (1.8)
other Noct.	10.6	10 (4.5)	9.2	12	7.9 (1.6)	10.8 (5.1)

**Table 4.** Total and mean number of species (species/experiment) caught by different types of semiochemical baited traps in the Hajdúság and ratio (%) of species according to their food source and protection (Prot.) and pest status. Vulnerable category contains species with faunistical importance and/or geographical interest, Str. pr. = Strictly protected. Small letters show significant differences between bait types, based on the Mann-Whitney U test (p < 0.05). IamOH: isoamyl alcohol-based, PHEN: phenylacetaldehyde-based.

		**Species**	**Species/exp.**	**Species**		**Species/exp. (± SD)**	
	**Volatile**		**(± SD)**	**IamOH**	**PHEN**	**IamOH**	**PHEN**
		**Number of species**					
**Food**	Herbaceous	118	34.9 (11.7)	79	103	43.0 (8.2)	31.8 (11.6)
	Arboreal	52	9.2 (9.0)	44	32	20.6 (7.8) a	4.8 (4.4) b
	other	9	1.6 (1.4)	7	7	2.4 (1.1)	1.2 (1.4)
**Prot.**	Total	16	2.6 (2.3)	14	11	5.0 (2.5) a	1.6 (1.4) b
	Vulnerable	14	2.3 (2.1)	12	10	4.6 (2.1) a	1.5 (1.3) b
	Protected	2	0.2 (0.4)	2	1	0.4 (0.5)	0.2 (0.4)
	Str. pr./N2000	0	0.0 (0.0)	0	0	0.0 (0.0)	0.0 (0.0)
**Pests**	Total	31	14.2 (4.5)	26	29	18.6 (1.7) a	12.5 (4.0) b
		**Ratio (%)**					
**Food**	Herbaceous	65.9	79.4 (13.3)	60.8	72.5	65.4 (7.5) a	84.8 (10.8) b
	Arboreal	29.1	17.8 (3.1)	33.8	22.5	30.9 (8.9) a	12.7 (10.8) b
	other	5	2.9 (2.6)	5.4	4.9	3.7 (1.6)	2.5 (2.8)
**Prot.**	Total	8.9	5.0 (3.1)	10.8	7.7	7.4 (3.0) a	4.1 (2.7) b
	Vulnerable	7.8	4.6 (3.0)	9.2	7	6.9 (2.4) a	3.7 (2.8) b
	Protected	1.1	0.5 (1.1)	1.5	0.7	0.5 (0.7)	0.5 (1.3)
	Str. pr./N2000	0	0.0 (0.0)	0	0	0.0 (0.0)	0.0 (0.0)
**Pests**	Total	17.3	32.6 (5.9)	20	20.4	28.8 (4.6)	34.1 (5.8)

**Table 5.** Ratio (%) and mean ratio per experiment (± SD) of the faunal types in the whole sample and in samples caught by semi-synthetic and synthetic lures in the Hajdúság. Small letters show significant differences between bait types, based on the Mann-Whitney U test (p < 0.05). IamOH: isoamyl alcohol-based, PHEN: phenylacetaldehyde-based.

	**Species**	**Species/exp.**	**Species**		**Species/exp.**	
	**Whole sample**		**IamOH**	**PHEN**	**IamOH**	**PHEN**
Holarctic	1.7	2.5 (1.5)	1.5	2.1	1.3 (0.8) a	3.0 (1.4) b
Extra-Palearctic	5.0	6.4 (2.7)	5.4	5.6	6.9 (1.9)	6.2 (3.0)
Euro-Siberian	53.6	67.6 (6.8)	54.6	57	60.1 (6.0) a	70.5 (4.5) b
Boreo-Continental	10.1	5.5 (3.8)	8.5	9.9	6.4 (2.1)	5.1 (4.3)
Holo-Mediterranean	24.6	15.8 (4.6)	23.1	23.2	20.3 (2.6) a	14.0 (4.0) b
Holo-Mediterranean-W-Asian	1.1	0.5 (0.8)	1.5	0.7	1.3 (0.8)	0.3 (0.7)
Ponto-Caspian-Manchurian	1.1	0.4 (1.2)	1.5	0.7	0.6 (0.8)	0.4 (1.4)
Ponto-Mediterranean	2.8	1.2 (1.8)	3.8	0.7	3.1 (2.2) a	0.4 (1.0) b

**Table 6.** Ratio (%) and mean ratio per experiments (± SD) of the ecological types in the whole sample and in samples caught by semi-synthetic and synthetic lures in the Hajdúság. Small letters show significant differences between bait types, based on the Mann-Whitney U test (p < 0.05). IamOH: isoamyl alcohol-based, PHEN: phenylacetaldehyde-based.

	**Species**	**Species/exp.**		**Species**			**Species/exp.**	
	**whole sample**			**IamOH**	**PHEN**		**IamOH**	**PHEN**
Altoherbosa	3.9	8.7 (6.6)		2.3	4.9		1.7 (1.6) a	11.3 (5.7) b
Arundiphilous	2.8	0.7 (1.4)		1.5	2.8		0.8 (0.8)	0.6 (1.6)
Betulo-Alnetal	1.1	0.5 (0.9)		1.5	1.4		1.1 (1.1)	0.3 (0.8)
Eurytopic	24.0	37.3 (7.9)		26.9	28.2		33.5 (2.2) a	38.8 (8.9) b
Marshy	3.9	1.9 (2.9)		3.8	4.2		1.4 (2.0)	2.2 (3.2)
Mesophilous	3.9	3.6 (1.8)		3.1	4.2		3.5 (0.9)	3.6 (2.1)
Nemoral	1.1	0.3 (0.7)		0.8	1.4		0.3 (0.6)	0.3 (0.8)
Populo-Salicetal	6.7	4.8 (3.2)		7.7	4.2		7.5 (2.8) a	3.8 (2.9) b
Psammophilous	0.6	0.5 (0.8)		0.8	0.7		1.0 (0.9)	0.3 (0.7)
Quercetal	12.8	6.5 (5.6)		15.4	9.9		12.5 (2.6) a	4.2 (4.7) b
Silvicol	17.9	19.7 (6.3)		21.5	16.2		25.5 (2.9) a	17.5 (5.8) b
Steppic	12.8	7.4 (4.4)		6.9	13.4		4.7 (2.3)	8.4 (4.7)
Migratory	5.6	7.3 (3.7)		5.4	6.3		5.6 (2.8)	8.0 (3.9)
Lichenophagous	2.8	0.8 (1.4)		2.3	2.1		0.9 (1.4)	0.7 (1.5)

**Table 7.** List of the most common species (mean constancy > 75%) of the Hajdúság, based on its spatial constancy in sites sampled with traps baited with isoamyl alcohol-based (IamOH) and phenylacetaldehyde-based (PHEN) lures with the food source, status faunal type and ecological type of species. Herb. = feeding on herbaceous plants, Arbor. = feeding on arboreal plants; BorCont = Boreo-Continental, EuSib = Euro-Siberian, ExtraPal = Extra-Palearctic, HoloMed = Holo-Mediterranean, PopSal = Populo-Salicetal.

	**Constancy**						**Ecological **
	**mean**	**IamOH**	**PHEN**	**Food**	**Status**	**Faunal type**	**type**
*Hadula trifolii*	96.2	100.0	92.3	Herb.	Pest	EuSib	Eurytopic
*Agrotis segetum*	96.2	100.0	92.3	Herb.	Pest	EuSib	Eurytopic
*Noctua pronuba *	92.3	100.0	84.6	Herb.	Pest	HoloMed	Eurytopic
*Macdunnoughia confusa*	86.2	80.0	92.3	Herb.	Pest	EuSib	Eurytopic
*Lacanobia oleracea*	84.6	100.0	69.2	Herb.	Pest	EuSib	Eurytopic
*Mythimna vitellina *	84.6	100.0	69.2	Herb.		EuSib	Eurytopic
*Mesapamea secalis*	84.6	100.0	69.2	Herb.		BorCont	Silvicol
*Mamestra brassicae*	80.8	100.0	61.5	Herb.	Pest	EuSib	Eurytopic
*Agrotis exclamationis*	80.8	100.0	61.5	Herb.	Pest	EuSib	Eurytopic
*Mythimna albipuncta*	76.9	100.0	53.8	Herb.		EuSib	Mesophilous
*Agrotis ipsilon *	76.9	100.0	53.8	Herb.	Pest	ExtraPal	Eurytopic
*Xestia c-nigrum*	76.9	100.0	53.8	Herb.	Pest	EuSib	Eurytopic
*Autographa gamma*	76.2	60.0	92.3	Herb.	Pest	Holarctic	Migratory
*Scoliopteryx libatrix *	73.1	100.0	46.2	Herb.	Pest	EuSib	Pop-Sal
*Thalpophila matura*	73.1	100.0	46.2	Herb.		EuSib	Silvicol
*Helicoverpa armigera*	72.3	60.0	84.6	Herb.	Pest	ExtraPal	Migratory
*Catocala nupta*	69.2	100.0	38.5	Arbor.		EuSib	Pop-Sal
*Agrochola circellaris*	66.9	80.0	53.8	Arbor.	Pest	EuSib	Silvicol

## Hosting institution

University of Debrecen
